# E-cigarette or Vaping Product Use-associated Lung Injury: A Case of an Adult Female Leading to Hospitalization

**DOI:** 10.7759/cureus.6765

**Published:** 2020-01-24

**Authors:** Jeffrey A Miskoff, Moiuz Chaudhri

**Affiliations:** 1 Medicine, Hackensack Meridian School of Medicine at Seton Hall University, Nutley, USA; 2 Internal Medicine, Shore Pulmonary, Ocean, USA

**Keywords:** vaping, evali, nicotine inhalation, tetrahydrocannabinol, smoking, marijuana

## Abstract

E-cigarette or vaping is an alternative to traditional cigarette use with potentially devastating consequences. The most recent update from the Centers for Disease Control and Prevention reports a total of 2,561 cases of vaping associated pulmonary injury as of December 27, 2019. This case described a 41-year-old female who presented with a clinical picture suggesting of bronchitis; however, a diagnosis of e-cigarette or vaping product use-associated lung injury was made.

## Introduction

Vaping is the inhalation of vapors created by electronic cigarettes (e-cigarettes) or similar vaping devices. These are battery-powered smoking devices that need cartridges filled with a liquid (flavored or unflavored) containing nicotine and other chemicals [1]. The liquid is heated into a vapor, which is inhaled by the user. Some people use e-cigarettes to inhale marijuana, tetrahydrocannabinol (THC) oil, and other chemicals [2]. Although there are different kinds of vaping e-cigarettes, the most common type is Juul™ (JUUL Labs, Inc., San Francisco, CA). It looks similar to a universal serial bus (USB) flash drive and can be charged using the USB port [2]. Generally, this type creates less smoke than other e-cigarettes; thus, it can be hard to identify [3]. Dank vape is a counterfeit THC-containing device, and the most commonly reported brand used especially, in the Northeast and Southern United States [3]. The purpose of this case is to increase awareness of e-cigarette or vaping product use-associated lung injury (EVALI) by reporting a case with respiratory, gastrointestinal, and constitutional symptoms combined with abnormal chest imaging. Her treatment included supportive management, systemic steroids, and antibiotics, the typical treatment described in EVALI patients. 

## Case presentation

**Figure 1:**
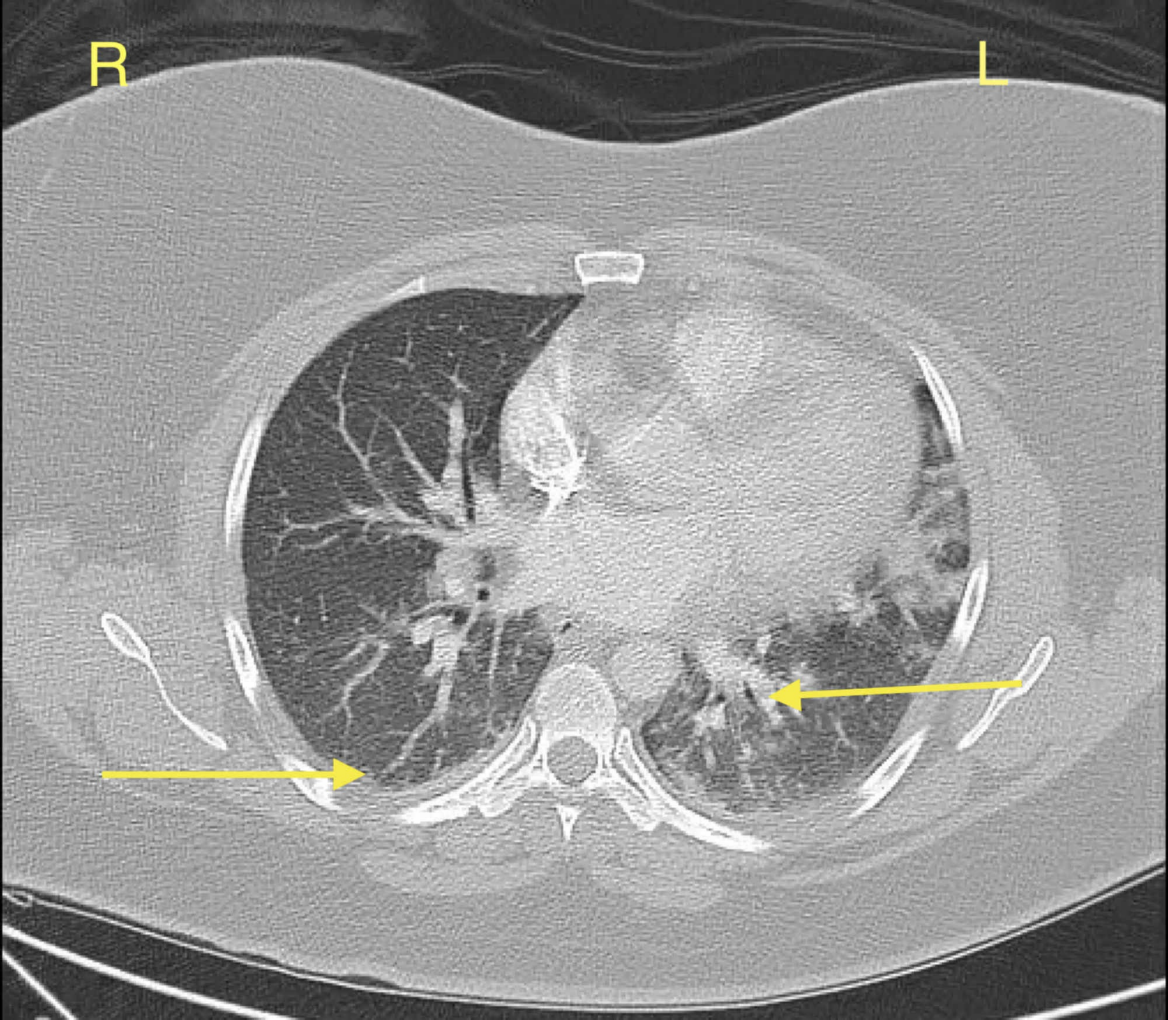
Ground-glass opacities in the left upper and right middle lobes.

We present a case of a 41-year-old female who presented to the emergency department (ED) on November 24, 2019, for a cough with brown and green colored sputum and wheezing. Chest x-ray (CXR) was identical to her previous CXR and did not reveal pneumonia-like respiratory processes. The patient was prescribed prednisone and azithromycin, and was later discharged as her acute condition stabilized. The next day, November 25, 2019, the patient presented with shortness of breath, nausea, vomiting, fever, chills, generalized malaise, arthralgia, and inspiratory crackles in the right and left lower lung fields. At this time, pulmonary consultation was requested, and the patient revealed that she was an active smoker, one pack-year, and switched to vaping about four to eight weeks before presenting to the ED. According to the patient, she was using THC and nicotine concomitantly for one month. She admitted to using Juul and Blu brand™ (blu eCigs, Charlotte, NC) for nicotine inhalation but was not sure of the specific brand of THC-containing device she tried in the past. The patient underwent CXR and a chest computed tomography (CT) angiogram, which were remarkable for pulmonary congestion and ground-glass opacities in the left upper lobe and lingula with minimal involvement of the right middle lobe, respectively (Figure 1). 

Physical exam revealed decreased air entry bilaterally and abdominal tenderness localized to the epigastric region. The patient was closely monitored and later discharged on December 2, 2019, with stable respiratory status. The patient was counseled on the recent outbreak of lung injuries and side effects associated with vaping. Furthermore, the patient was encouraged to stop vaping and was encouraged to follow up with a pulmonologist. 

## Discussion

EVALI is classified as an acute respiratory illness that can be life-threatening [4]. Vaping can be traced as far back as the 1930s; its addition as an alternative to traditional cigarettes took place in 2003 with the help of a Chinese pharmacist, Hon Lik [5]. In 2007, electronic cigarettes entered the United States marketplace. Initially, it was sold online without specific regulations. Its popularity increased when companies such as Reynolds American and Lorillard made it available in the stores. Data suggest that in 2012, approximately 1.78 million students, grades 6-12, had tried the product [6,7].

According to the data, more than 2,000 cases of EVALI have been reported to the Centers for Disease Control (CDC) in the last few months, summer 2019 to December 3, 2019 [8,9]. Data from the CDC suggest that the majority of the reported cases include males and below the age of 35 years [4]. Patients diagnosed with EVALI present with symptoms typically seen in acute fibrinous pneumonitis, diffuse alveolar damage, and a mixture of other disease processes such as alveolar hemorrhage, lipoid pneumonia, and acute eosinophilic pneumonia. According to the reports, nicotine, THC, cannabinoid oils are among the most common ingredients behind EVALI [4,9]. Analysis suggests that 80% and 54% of cases reported to the CDC involved the use of THC and nicotine products, respectively. Furthermore, 40% of cases involved the use of THC and nicotine concomitantly. In addition, vitamin E acetate, a synthetic form of vitamin E, has been found in bronchoalveolar lavage of patients with EVALI. Majority of the patients present with shortness of breath, cough, chest pain, hemoptysis, and gastrointestinal symptoms with rapid progression to respiratory failure [4].

The patient presented to our care on November 25, 2019, with clinical symptoms suggesting pneumonia, which did not respond to traditional pharmacologic management. Later, a chest CT was remarkable for new ground-glass opacities. Her symptoms in conjunction with recent vaping along with reported EVALI cases suggest this patient might be suffering from vaping-induced respiratory complaints. Her treatment included supportive management, systemic steroids, and antibiotics, typical treatment described in EVALI patients. She improved clinically, and was discharged with an instruction to undergo a pulmonary follow-up as soon as possible. 

## Conclusions

EVALI epidemic has blown into a full-blown public health crisis forcing CDC to provide regular updates encouraging healthcare providers to be cognizant of clinical presentation. It is important to ask the patient about smoking especially vaping. Lastly, educating patients and their families about this potentially lethal condition despite not being strictly regulated is of the utmost importance.
